# Herman Boerhaave’s Clinical Teaching: A Story of Partial Historiography

**DOI:** 10.1007/s11673-023-10244-9

**Published:** 2023-04-04

**Authors:** Patrick J. Fiddes, Paul A. Komesaroff

**Affiliations:** 1 Monash University Faculty of Medicine, 124/461 Saint Kida Rd., Melbourne, Victoria 3004 Australia; 2 Monash University Faculty of Medicine, 10 Barnato Grove, Armadale, Victoria 3143 Australia

**Keywords:** Boerhaave, Innovative, Clinical teaching, Post-mortem, Leiden, Lindeboom

## Abstract

Gerrit Lindeboom’s biography, *Herman Boerhaave: The Man and His Work*, presents a heroic account of Herman Boerhaave’s life and his many contributions to medicine and medical education. He is portrayed as an outstanding eighteenth century educator who introduced into Leiden’s Medical School a novel method of clinical teaching that was to be widely adopted and today remains at the centre of medical student instruction. Lindeboom’s historiography induced a resurgence of interest in Boerhaave, a renewal of the myth concerning Boerhaave’s innovative teaching and the publication of many acclamatory articles and false epithets, and several critical analyses. Such varying responses prompted this critical examination of the extant Boerhaavian literature, an appraisal of Lindeboom’s objectivity and an assessment of his representations of Boerhaave’s clinical teaching. In doing so, the moral nature of his historiography and that of those who were to sustain his assertions will be established, and the myth that surrounds the novelty and excellence of Boerhaave’s clinical teaching will be evident.

*Simplex veri sigillum* 1

## Introduction

*The course of human history is determined no more by what is true than by what men believe to be true* (Dunning 1914, 220).

Herman Boerhaave (1669–1739) was an extraordinarily accomplished and capable polymathic physician, a Calvinist theologian and philosopher, a polyglot, historian, mathematician, iatrochemist, botanist, and advocate for Cartesian mechanistic theories whose contributions to the advancement of chemistry, botany, and the teaching of medicine are legendary. He was appointed to four Leiden University Medical professorships, was twice Vice Chancellor of Leiden University and was elected to both the Academy of Sciences of France and the Royal Society of London. His renown for the excellence of his lecturing, his many publications and his skill as a physician were enhanced by his students who advanced teaching in many European cities and in Edinburgh.

Boerhaave’s high regard was perpetuated through the autobiographical notes he left that were employed in the writing of his funeral eulogy and were to facilitate the writing of subsequent biographies and commentaries concerning his life and his work, which established the myth that has surrounded his clinical teaching. That myth was revitalized by Lindeboom in his 1968 biography, *Herman Boerhaave: The Man and His Work*, in which Lindeboom made four assertions: Boerhaave brought together the theories and basic sciences of medicine; he systemized Leiden University’s medical curriculum; he introduced a novel method of clinical teaching, and, he changed the course of medical student teaching.

Lindeboom’s partisan biography excited an ardent supportive literature, several dubious epithets such as “Father of Bedside Teaching” and several scholarly questionings of his historiography. The nature of such epithets and the partiality exhibited by his acolytes prompted the undertaking of this inquiry into Lindeboom’s historiography, the ethicality of his presuppositions and assertions, and whether his portrayal of Boerhaave’s contributions to medicine’s clinical teaching will establish if the error inherent in “what men believe to be true” may be applied to Lindeboom’s partisan biography.

Lindeboom as Professor of Medicine at Amsterdam Free University, had published *Bibliographia Boerhaaviana* in 1959, *Iconographia Boerhaavii* in 1963, three volumes of letters between Boerhaave, his colleagues and his students as *Boerhaave’s Correspondence*, and *Boerhaave and his Time*, when writing *Herman Boerhaave: The Man and his Work*, could be expected to have complied with the historiographer’s inherent moral responsibilities: to critically appraise all available data; to determine its truth and present it objectively; to accept his moral and intellectual responsibility to do justice to his data; to control his sympathies and presuppositions, and, to make objective value judgements on the material he employed to ensure the trustworthiness of his writing.

In undertaking this enquiry “clinical teaching” will be taken to represent the teaching of the semiotic skills and cognitive processes that are required to establish each patient’s diagnosis and her management, that takes place in a hospital or medical clinic and is taught at the patient’s bedside or in her presence. And, “semiotics” will be regarded as the study and interpretation of each patient’s symptoms and signs.

## Part 1. A Precis of Boerhaave’s Medical Training, His Appointments and His Teaching Responsibilities

Herman Boerhaave was born when the ideas and discoveries of men such as Boyle, Descartes, Huygens, Locke, Malpighi, Newton, Willis, Vesalius, and van Leuwenhoek were changing the science of medicine and its teaching. He was an Enlightenment man, a product of his environment, who as the “right man at the right time,” is remembered and revered as a great physician, who, “came closest to the medical ideal of [his] time” (Lindeboom 1968a, 7).

During his final illness Boerhaave prepared the autobiographical notes that his friend Albert Schultens used in the writing of Boerhaave’s eulogy which both Samuel Johnson and William Burton relied on in writing their Boerhaave biographies, as did other Boerhaave students and Gerrit Lindeboom.

Following his 1690 graduation from Leiden University with a doctorate in philosophy and theology, Boerhaave commenced his heutagogic study of two millennia of medical texts, attended Nuck’s 1691 annual winter anatomical dissection, visited various anatomical museum collections and performed animal vivisections. Such studies gave Boerhaave a knowledge of medicine’s history, of its theories and practices, and an appreciation of anatomy without having had either supervision or guidance:

The whole of Boerhaave’s medical studies must have been completed in the period lasting from the beginning of 1691 until the middle of 1693 … Moreover, the way he arranged his studies was most uncommon and showed that he had learned to work completely independently. It is an established fact that he did not attend any medical lecture at the University of Leyden. As a physician Boerhaave was almost totally self-taught. (Lindeboom 1968b, 28)

In July 1693, Boerhaave gained his doctorate of medicine from the smaller and less well-regarded Harderwijk University Medical School that had one professor and few students (Lindeboom 1968b, 40). He enrolled, was examined, and the next day defended his thesis; *De utilitate explorandorum excrementorum in ægris, ut signorum*. By achieving an MD through this process, Boerhaave chose to make a moral decision that a career in medicine could best be achieved through an intellectual and theoretical process of learning rather than by pursuing a degree at Leiden’s highly-regarded and clinical-centred Medical School. His decision would characterize the way in which he would teach medicine as an academic.

Shortly after Boerhaave’s Harderwijk graduation he began to see patients, and while he “practiced … with not so many patients” (Lindeboom 1968c, 32), supported himself by teaching mathematics (Lindeboom 1968b, 47). In 1701 despite his lack of training and limited medical experience, Leiden University finally agreed to appoint him Lecturer in the Institutes of Medicine. Lindeboom commented on the complex negotiations that led to Boerhaave’s appointment, (Lindeboom 1968b, 50–51) about which Harold Cook wrote; “The notion that he became a professor … because his genius was recognised has been demolished by Maarten Ultee’s account of how he finally obtained the post” (Cook 2000, 223).

Boerhaave’s appointment required him to lecture matriculant (enrolled students) on subjects of physiology, pathology, semiotics, therapeutics, and hygiene for four days each week had been taught through much of the seventeenth century, and were the contents of Lazarus Riverius’ 1655 *Institutiones Medicae*. As well, Boerhaave was entitled to lecture non-matriculant fee-paying private attendees and lectured on the Institutes, chemistry, and anatomy, which he soon discontinued (Lindeboom 1968c). In 1702, Lindeboom commenced the private teaching of practical medicine (*praxis medica* or practice of medicine) and in 1707, six years after commencing Institutes teaching, he published *Institutiones medicae*, that concentrated predominantly on physiology and to a lesser extent, pathology (Lindeboom 1968b, 70–72). The next year he published *Aphorismi de Cognoscendis et Curandis Morbis* containing the 1479 aphorisms he employed in his private *praxis medica* lectures. Each book was derived from the works of others, and in the *Aphorismi* he explained its source in the preface:

The Industry of the Ancient Greeks, the Diligence of the Succeeding Arabians, and the Exactness of a few among the late Moderns, have supplied us with Experiments altogether necessary to the finishing of this Work. But Anatomy and Mechanics, both better and more universally understood in our Day have laid the Foundations and Spun the Thread of our Reasonings. (Boerhaave 1986, 3)

Boerhaave was appointed Professor of Medicine and Professor of Botany in 1709 and in 1714, Professor of the Practice of Physick and Physician to the Saint Caecilia Hospital. That appointment gave Boerhaave his first experience of hospital medical practice and clinical teaching, and his reading-acquired theory-based aphorisms became the basis for his *praxis medica* clinical lecture-demonstrations to the matriculant students.

Boerhaave’s lectures became very popular, for “as a good teacher, Boerhaave spoke slowly so that his hearers could understand and write down his Latin sentences,” (Lindeboom 1968b, 58) and, as his teaching reputation grew, so too did his private student numbers and his standing among his colleagues in The Netherlands, across wider Europe, and in Britain (Lindeboom 1974, 16).

## Part 2. A Survey of Boerhaavian Historiography Between 1739–1765, 1766–1928, and 1968–2009

The story of Boerhaave’s clinical teaching will be reviewed through three distinctive periods of extant Boerhaavian historiography that comprise: five biographies and the commentaries of two famous students; the judgements by professional or medical historians on the merit of Boerhaave’s teaching, and the literature that Gerrit Lindeboom’s biography generated.

### i. 1739–1765: A Boerhaave Eulogy and Five Biographies

During Boerhaave’s final weeks he composed an autobiography that his friend Albert Schultens used in his eulogy, *Oratio academician memoria Herman Boerhaave*. Schultens “discussed fully the life and works of Boerhaave, and expiated broadly on the virtues of his mind and on the devoutness of his heart” (Lindeboom 1968b, 220). It was first published in the *Bibliotheque Raisonnée* in 1741 and later published in French, Dutch, and English. Boerhaave’s notes were subsequently collated and published by William Burton as *Commentariolus de familia, studiis, vitae cur-su, &c. propria Boerhaavii* (Burton 1743, 203–213), which he later translated into English and published in London in 1749.

In 1739, Samuel Johnson’s biography, *Herman Boerhaave*, was published in four successive essays (Johnson 1739), among which was this comment that was first noted by Schoneveld, 2020):

The following account of the late Dr. Boerhaave, so loudly celebrated, and so universally lamented through the whole learned world, will, we hope, be not unacceptable to our readers; we could have made it much larger, by adopting flying reports, and inserting unattested facts: a close adherence to certainty has contracted our narrative, and hindered it from swelling to that bulk, at which modern histories generally arrive. (Johnson 1739, 270)

Johnson followed Schultens’ oration closely and portrayed Boerhaave in heroic terms and likened him to Socrates, wrote; “It is, I believe, a very just observation, that men’s ambition is generally proportioned to their capacity. Providence seldom sends into the world with an inclination to attempt great things who have not abilities likewise to perform them” (*ibid*, 275). Johnson included two extracts from Schultens oration:

It is perhaps incredible that he did not attend any lectures by any professor of medicine … In 1714, he was made physician to St Augustine’s hospital … into which the students are admitted twice weekly to learn the practice of physick … This was of equal advantage to the sick and the students, for the success of his practice was the best demonstration of the soundness of his principles … (*ibid*, 281)

And,

Of his sagacity, and the wonderful penetration with which he often discovered and described, at the first sight of the patient, such distempers as betray themselves by no symptoms to the common eyes, such wonderful relations have been spread over the world, as, though attested beyond doubt, can scarcely be credited. (*ibid*, 311)

While not using flying reports and unattested facts, Johnson implied that they had existed.

William Burton’s 226 page biography, *An Account of the Life and Writings of Herman Boerhaave* was published in 1743 and remained the most reliable account of Boerhaave’s life and work for two centuries. Burton, in his account of Boerhaave’s life and virtues “made massive use” of Schultens’ *Oratio *(Lo Presti 2005, 475), and noted both Johnson’s biography and Bernard de Fontenelle’s 1741 *Eulogy of Professor Boerhaave* (Burton 1743, ii). Burton comprehensively discussed Boerhaave’s education, his undergraduate academic studies, his self-directed pursuit of medical studies and chemistry, and his writings, and, in quoting Boerhaave’s penultimate notes, wrote; “he opened the bodies of animals; and was sedulous in his attendance in the theatre at the anatomical dissections of the celebrated Nuck” (Burton 1743, 14). However, As Nuck had died in August 1692, Boerhaave could have attended only the winter 1691 dissection. Burton then discussed Boerhaave’s lecturing and, in some detail, his *Institutiones* and *Aphorismi*, his extensive writings, orations, and public lectures. His report of Boerhaave’s careful examination of patients and formulation of therapies at the *collegium medico practicum* (Burton 1743) must have been based on the observations of others, for Burton as a Leiden student from 1724–1730 (de Vreugd 2022) had registered after Boerhaave had ceased *collegium* teaching. However, Burton did not include *collegium* post-mortems in their accounts (Knoeff and Zwijnenberg 2016, 88) despite that Lindeboom asserted that Boerhaave had undertaken many (Lindeboom 1968b). Burton’s further contribution was his compilation of the *Commentariolus.* In its Preface, he wrote;

It was not without reason expected that the veneration many in neighbouring countries retained for his memory, [would] before have been manifested in a distinct volume especially since his funeral oration by the learned and reverend professor Schultens has applied material for one. (Lindeboom 1968b, 106)

He concluded his biography by writing:

This great man is departed to the irretrievable loss of philosophy and physick: long was he the physician of all Europe. Never was praeceptor more beloved, more celebrated nor physician more consulted; he arrived to eminence in all the several branches of medicine, had the glory of teaching them with equal applause, and the happiness of feeling himself admired without being obnoxious to the effects of envy or to disparaging contradiction; insomuch that he was never mentioned by the greatest of his contemporaries but with encomiums. His sole authority without the support of arguments was admitted as decisive. He is no less successful in practice than learned in theory, and is therefore styled the *Batavian Hippocrates*. The qualities of his mind have rendered him still more amiable, than those of his understanding. He was a sure patron to men of learning and genius, employing his own reputation as it were wholly for their service. (Burton 1743, 73–74)

Jean Offray de La Mettrie, a 1733 Leiden student, published the third biography, “Vie de M. Herman Boerhaave” (Scholten 2011) as a preface to his 1743 French translation of Boerhaave’s *Institutiones*. He also translated Boerhaave’s Aphorisms, Materia Medica, Chemical Proceedings, Chemical Theory, and the Institutions into French. But, in his subsequent 1747 acerbic book, *L’Homme Machine*, he stated; “Willis and Perrault [were] careful observers of nature, whereas nature was known to the famous Leyden professor only through others and second hand, so to speak” (La Mettrie 1912, 138). While Lindeboom dismissed this first criticism of Boerhaave’s heutagogic learning, he noted other comments made by Mettrie (Lindeboom 1968b).

Mathew Maty, a Leiden student from 1732–1740 and Principal Librarian at the British Museum wrote the fourth biography, “Essay on the Character of the Great Doctor, or Critical Praise of Mr. Herman Boerhaave” in 1747. Lindeboom employed two quotations from Maty’s essay that illustrate Lindeboom’s bias. The first showed Boerhaave to be a good doctor;

Many times had he not declared to those who came to consult him that he saw their pain; that his art did not furnish him with a remedy for their infirmities; that he was careful not to undertake a cure, to which he saw no chance of success (*ibid*, 313)

whereas the next, “People complain that he had neither enough politeness nor even enough respect for his patients. He gave them, it is said, little welcome, received them with a dry eye, dismissed them abruptly” (ibid, 314), caused Lindeboom to refer to Maty as one who had envied, criticised and belittled Boerhaave (*ibid*).

While the fifth biography by Chevalier de Jaucourt, “Vie de Boerhaave” remained unnoticed in an encyclopaedia, neither of Boerhaave’s most two famous students, Albrecht von Haller and Gerard van Swieten, published biographies. However, each revised and edited Boerhaave’s textbooks.

Haller studied medicine at Tübingen in 1724–1725 and Leiden in 1725–1727, graduating when aged eighteen. His revision of Boerhaave’s *Institutiones* was published in seven volumes from 1739–1744 as *Boerhaave’s Proper Institutes of Medicine*, and in 1751 he published *Hermanni Boerhaave Methodus Studii Medici Emaculata* and its index in 1759 (von Haller 1745; Boerhaave 1759). Two of Haller’s letters reveal his high regard for Boerhaave; “my beloved praeceptor, a man of refined taste and a speaker or lecturer so logical and charming that one more gifted can be hardly imagined” (Butt 1917, 445) and, in a 1780 letter to his daughter,

Fifty years have almost elapsed since I was the disciple of the immortal Boerhaave: but his image is always in my mind. I have always before my eyes the venerable simplicity of that great man, who possessed to an eminent degree, the talent of persuading. (Knoeff 2010, 283)

Two other letters reveal his concerns:

Von Haller blamed Boerhaave for not having kept up with the latest developments in medicine during the last twenty years of his career. He was also very critical of the, in his eyes, seriously deficient anatomical and physiological knowledge of his master.(*ibid*, 279)

And,

Mr van Swieten, inseparably attached to his master, has adopted all his systems and hypotheses. Mr Haller, full of veneration for the same master, admits however only those which he considers right, and he opposes—although with respect—to the smallest brilliant error which could blind him. (*ibid*)

Neither of these criticisms was included by Lindeboom.

Swieten also graduated in Leiden medicine in 1725 and continued to attend and record Boerhaave’s *praxis medica* lectures for “twenty years” (Lindeboom 1968b,191). His notes, with additions, were published in twelve volumes from 1742–1747 as *Commentaries upon the Aphorisms of Dr Herman Boerhaave* (Welcome Library 2020). Swieten explicated Boerhaave’s *Aphorismi* and corrected the criticisms of its brevity and obscurity(*ibid*).

Given Boerhaave’s retirement from *collegium clinical* teaching in 1723, and the negligible number of *collegium* admission after 1714, (*vide infra*, 20–21), neither Burton, Haller, nor Swieten would have experienced Boerhaave’s *clinical* teaching; their *praxis medica* lectures would have been without patients.

Of these biographies and commentaries, Johnson’s inferences, La Mettrie’s comments, and Haller’s criticisms were each dismissed by Lindeboom, and Maty’s criticism was rejected. As each was unfavourable to Lindeboom’s thesis, his selective use of data has brought into question his historiographic bias.

### ii. 1766–1928: Judgements About Boerhaave’s Teaching

Boerhaave’s system of clinical teaching and his textbooks were introduced in the Edinburgh Medical School from the 1720s by professors who had been Boerhaave’s students, whereas William Cullen, Professor of the Institutes of Medicine in 1766 and the Practice of Medicine in 1773, had not been a Leiden student. As Professor of Physic at Glasgow, Cullen in 1747 criticized Boerhaave’s textbooks and determined not to lecture in Latin (Significant Scots), which he also did in Edinburgh. In 1775, he published a four volume textbook of practical medicine, in which he made the first substantial criticism of Boerhaave’s clinical teaching based on the theories presented in his book *Aphorismi Decognoscendis et Curandis Morbis.*

Whoever will consider the merits of Dr. Boerhaave, and can compare his system with that of former writers, must acknowledge that he was very highly esteemed, and gave a system which was at that time deservedly valued, but, in the progress of an inquisitive and industrious age, it was not to be expected that any system should last so long as Boerhaave’s has done. The elaborate Commentary of Van Swieten on Boerhaave’s system of practice, has been only finished a few years ago, and though this Commentator has added many facts, and made some corrections, he has not made any improvement in the general system. It is even surprising that Boerhaave himself, though he lived near forty years after he had first formed his system, had hardly in all that time made any corrections of it or additions.

When I first applied to the study of Physic, I learned only the system of Boerhaave, and even when I came to take a Professor’s chair in this university, I found that system here in its entire and full force; and as I believe it still subsists in credit elsewhere, and that no other system of reputation has been yet offered to the world, I think it necessary for me to point out particularly the imperfections and deficiencies of the Boerhaavian system. In order to show the propriety and necessity of attempting anew to execute this, however, so fully as I might, would lead me into a detail that can hardly be admitted of here; and I hope it is not necessary, as I think, that every intelligent person, who has acquired any tolerable knowledge of the present state of our science, must, in many instances, perceive its imperfections. (Cullen 1775, 33–35)

A further criticism was made by Jean-Eugene Dezmimeris in 1837 in his *Dictionaire Historique de la Medicine Ancienne et Moderne*. He wrote that Boerhaave’s system had persisted through the works of his students, Haller, De Haens, and van Swieten, “who filled the eighteenth century with the glory of his name” (Dezmimeris 1837, 15–17). Dezeimeris referred to the improvements that Haller made to Boerhaave’s *Institutiones* and Swieten made to the *Aphorisms*, each of which had perpetuated Boerhaave’s reputation.

And, on the centenary of Boerhaave’s death, a twelve-page memoir of Boerhaave was published by Thomas Pettigrew; after discussing Boerhaave’s life before his 1701 Lector appointment, and the history of the medical sciences contained in Boerhaave’s *Institutiones*, Pettigrew criticized both Boerhaave’s clinical experience and the formulation of his aphorisms.

His aphorisms would be almost unintelligible but for the commentary of Van Swieten, and would long since have been consigned to the “tomb of all Capulets.” They are formed upon gratuitous suppositions, for which no proof can be offered. They were the product of great reading and patient research; but they wanted the experience and judgement only to be obtained at the bed-side of the patient. Boerhaave altogether appears on the field of medicine rather as a lecturer or teacher than a practitioner: his comprehensive mind—his astute discrimination—his erudition—all combined to render him most popular as an instructor; and his renown must be considered as based upon the duties of his professorships at Leyden, rather than any particular acumen. (Pettigrew 1838–1840, vol 3, 53–65)

However, Lindeboom, neither in his ‘Index of Persons’ in *Herman Boerhaave* nor in *Medical Education* (Lindeboom and Ham 1974, 163–165), listed Cullen, Dezeimeris, Pettigrew nor Gerard Suringar, who, in 1860 published the first of eighteen articles on the history of Leiden’s clinical school and its teachers. The twelfth, in 1866, considered the clinical teaching that Herman Boerhaave and Oosterdijk Schacht had shared in teaching at the Saint Caecilia *collegium* (Suringar 1969, 199–227). Suringar relied on Haller for his accounts of Boerhaave’s *Institutes* lectures and Swieten for Boerhaave’s *collegium praxis medica* teaching. While focussing on Boerhaave’s lectures and practical teaching, Suringar commended he and Schacht, referring to them as “two famous men” and “great masters.” When discussing the *collegium* anatomical room, Suringar made no mention of Boerhaave post-mortems. In referring to Haller’s Boerhaave epithet, *Communis Europae praeceptor*, Suringar wrote:

With these words he seems to have intended less the content of the writings published by Boerhaave than his oral lectures themselves. Although his writings were also highly appreciated in other countries … it was the direct influence that Boerhaave exercised through his lectures in medicine and his example as a clinical teacher … that Haller alluded to … Among the many pleasant and favourable qualities that characterised Boerhaave’s personality was his excellent talent in teaching … he had a natural aptitude for this as was evident in the ease with which he spoke and in his pleasing and fascinating recitals … His listeners … appreciated not only the soundness of his teaching, but also his natural simplicity and uncluttered presentation. (*ibid* 2200–2201).

Suringar’s history of Leiden’s clinical teaching was criticized by Rina Knoeff, who wrote: “Suringar turned the medical faculty of Leiden University—as well as Herman Boerhaave—into a centre of excellence. More than Schultens ever did, Suringar emphasized Boerhaave’s practical medical [clinical] teaching, an interpretation that was followed by most medical historians up to the 1960s” (Knoeff 2010, 272).

Adrianus Maas’s 1866 eight page booklet “Herman Boerhaave” was next but remains untranslated in the Netherlands National Library and unlisted by Lindeboom.

Of greater significance were three criticisms by the famous French historian Charles Daremberg in his 1870 book, *Histoire des Sciences Medicales*. In reviewing the history of the Institute of Medicine subjects he argued that Boerhaave’s *Institutes* comprised, “some of the half-truths and almost all the errors of time [that] have come together” (Lindeboom 1968c, 36–37; Daremberg 1870, 896) and [Schultens’ oration was] “tiring with a convulsing and gasping enthusiasm. This Oratio begins, continues and ends with exclamation marks” (Daremberg 1870, Vol ll, 889). He concluded,

In the *Aphorisms* and in the *Institutes* there is nothing profound nor anything beyond the ordinary reach of the human mind; neither is the form new nor the doctrine sublime and innovative; it seems to me that the commentary by his disciple Van Swieten, is far better than the text of the master … (ibid, 890, quoted in Cook, 2000, 223).

Lindeboom quoted the criticisms on pages 889 and 896 of Daremberg’s *Histoire* but disregarded the third on page 890.

Daremberg was followed by William Lusk, who in 1895 wrote; “Boerhaave’s greatest glory was the prominence he gave to clinical instruction … he adopted the plan of examining a few patients...at the bedside” and [quoting Burton] “Long was he the oracle of his faculty. Never was preceptor more beloved, Professor more celebrated, nor physician more consulted” (Lusk 1895, 117).

He was followed in 1900 by Sir Clifford Allbutt, Cambridge Regius Professor, who criticized Boerhaave’s writings:

Boerhaave’s position in the first quarter of the eighteenth century has truly been called “stupendous”; perhaps no physician ever enjoyed so great a fashion with so little scientific merit. It is difficult to say wherein Boerhaave benefited medicine save in course by his pursuit of practical clinical teaching in hospital wards; wherein, however, he seems to me to have shown less insight and skill than his pupil van Swieten … However, Boerhaave, chiefly by virtue of his personal character, was a prodigious leader … and, in his writings at any rate, [seems] to have contented himself with hashing up partial truths and the entire errors of his time. (Allbutt 1900, 1849–500)

But, in 1907 his fellow Oxford Regius, Sir William Osler, gave his Hippocratist praise; “Boerhaave, the *Dutch Hippocrates*, under whom the objective methods of Sydenham reached its highest development” (Osler 1907, 8). And, in 1913, in that same vein said:

Under Boerhaave, this [clinical teaching] was so developed that to this Dutch university students flocked from all parts of Europe … he had a strongly objective attitude of mind towards disease, following closely the methods of Hippocrates and Sydenham. He adopted no special system … his clinical lectures, held bi-weekly, became exceedingly popular … and the cases were studied [with a] freedom from fanciful doctrines. He was much greater than his published work would indicate, and as is the case with many teachers of the first rank, his greatest contributions were his students. (Osler 1921, 193)

Then, in the more critical context of the 1917 meeting of the Historical Section of the Royal Society of Medicine, Osler had given a different judgement:

His clinical medicine is rescued from oblivion by one or two important observations, of which the most often quoted is the case of rupture of the oesophagus. What to his contemporaries and immediate successors appeared refined gold is dross to us, and his books on medicine are dead today. (Osler 1917, 31)

Also in 1917, Albert Buck wrote; “[Boerhaave] owed a large part of his fame to the admirable manner in which he conducted his clinical teaching” (Buck 1917, 439), and, regarding the *Aphorisms*:

This work is a very concise statement of the author’s views regarding pathology, anatomical pathology and therapeutics … I have not found it an easy matter to understand … if one wishes to ascertain what Boerhaave’s are … one should read … the Commentaries of Van Swieten. (*ibid*, 441)

In 1919, David Riesman expressed concern about the novelty of Boerhaave’s clinical teaching:

Until quite recently I accepted as fact, having seen it in a number of works, that the first clinical teacher was Boerhaave; but Renaudot, Petersen, Puschmann, and other reliable authors have clearly demonstrated that the credit for inaugurating clinical teaching belongs to two otherwise unknown Italians, Oddi and Bottoni. (Riesman 1919, 143)

Whereas, in 1927, the Italian historian Arturo Castiglioni wrote; “Boerhaave led clinical teaching … derived in a direct line from the school of Sydenham,” (Castiglioni 1941, 548) and as

… one who seeks to arrange the problems of nature in logical sequence and as an eclectic who brought the patient back to the centre of medical attention by teaching the examination of the patient first and then considering the disease in his construct of theories. (ibid, 616)

Of greater concern was Charles Singer’s unsubstantiated assertion in his book, *A Short History of Medicine*:

Boerhaave had very few beds at his disposal, but never did a man make better use of his opportunities. Beside clinical, chemical, botanical and anatomical instruction he followed his patients as died into the post-mortem room and there demonstrated to his students the relation of lesions to symptoms. He is thus the introducer of the method of medical instruction still in vogue in our modern medical schools. (Singer 1928, 140)

Singer’s assertion, like Lindeboom’s “Boerhaave must have carried out many [post-mortems],” (Lindeboom 1968b, 106) was based on supposition, for no previous author had suggested that he had performed post-mortems at the Saint Caecelia autopsy room.

By omitting the criticisms of Cullen, Pettigrew, Dezeimeris, Daremberg, Allbutt, and Riesman, Lindeboom’s avoidance of unfavourable data is again evident.

### iii. 1968–2009: Lindeboom’s Herman Boerhaave the Judgements by Five Historians

Little more was written about Boerhaave until 1959 when Lindeboom published *Bibliographia Boerhaaviana* and four further books to 1968 when he published *Hermann Boerhaave, The Man and his Work*. He was assisted in its preparation and editing by Edgar Underwood (1968a), who wrote its foreword and published “Boerhaave after Three Hundred Years,” (Underwood 1968b, 824) in which Underwood summarized Boerhaave’s life, his achievements, and influence, reviewed the previous century of Boerhaavian literature and criticized the “misconceptions” of both Allbutt and Osler. But, of more interest was his comment; “My brief search has disclosed amazing inaccuracies and inconsistencies … there is no agreement on the question of whether Boerhaave did, or did not, found a ‘system’” (*ibid*).

Lindeboom, by referring to Boerhaave’s “method” rather than his “system,” did not share Underwood’s uncertainty in writing:

Indeed, Boerhaave was, first of all, a teacher … It may be that Boerhaave … at least in medicine was more a transmitter and transmuter than a creator … Boerhaave’s influences on medical practice depended not so much on the interesting cases he demonstrated at his clinical lectures, but on the method he followed and taught … he raised the standard of bedside teaching to a hitherto unknown height. (Lindeboom 1968c, 36–38).

And Lord Cohen, in reviewing Lindeboom’s biography, matched Lindeboom’s assertion; “Boerhaave’s scholarship cannot be gainsaid … his fame as a clinician was legendary … As a clinical teacher, he was unsurpassed in his age … Boerhaave certainly shone as a bedside teacher” (Cohen 1969, 408–409).

A like assertion was made by a third historian, Frank Brechka:

While the facts of his life may be quite evident, the genius of Boerhaave and the springs of his reputation as the greatest physician of the eighteenth century are a bit more difficult to define. He made no revolutionary discoveries, offered no new theories, proposed no exceptional ideas. He was as unoriginal as any academician could be. Yet he was famous all over Europe … It was not his competence in either botany or chemistry that made Boerhaave great … It was, rather, in medicine that he made his most important contributions as a Great Teacher and a systematist at a time when a system was needed. (Brechka 1970, 58–59).

In contrast, the fourth historian, Harold Cook, doubted the objectivity of Lindeboom’s work:

Boerhaave’s is a name to reckon with … Our “knowledge” about Boerhaave’s importance, however, is often familiarity with the icon rather than the person. The mythology makes him into perhaps the greatest rational systematist of modern medicine, providing the foundation for eighteenth-century academic medicine … Until recently, most accounts of Boerhaave have taken their direction from the *Oratio* … by Schultens … [that] served as the basis for the biographical essays by Samuel Johnson and by Boerhaave’s student and admirer William Burton … They too saw him in heroic terms … More significantly Gerrit Lindeboom’s major … study is a revival of Schulten’s enthusiasm in modern dress. (Cook 2000, 221–222)

The fifth, Elizabeth Williams, in reviewing Mart van Lieburg’s 2007 revised second edition of *Herman Boerhaave*, described Lindeboom’s approach to medical biography as conventional, and wrote:

The changes that have marked medical history since the late 1960s have rendered the methods, interpretative concerns, and tone of Lindeboom’s biography in good part obsolete … While Lindeboom’s research was meticulous in regard to Boerhaave’s life and activities, he made no effort to contextualise his subject’s medical and scientific contributions … it is evident that Lindeboom drew personal inspiration from Boerhaave’s example … it does not serve as a serious critical study. (Williams 2009, 256)

Among these five historians Underwood, Cohen, and Brechka endorsed Lindeboom’s contentions regarding Boerhaave’s teaching methods, while Cook and Williams questioned the impartiality, and so the integrity, of Lindeboom’s historiography.

## Part 3. Boerhaave’s *Collegium* Teaching and Post-Mortems: A Review of Lindeboom’s Assertions

Two hundred and seventy years of Boerhaavian historiography has shown a partiality in Boerhaave’ extolment by Schultens, Burton, and Suringar, in Singer’s assertion that he was the greatest physician of modern times, in Cohen’s praise for clinical teaching that had been unsurpassed in his time, in Lindeboom’s contention that Boerhaave raised the standard of bedside teaching to a hitherto unknown height, in Underwood’s claim, “it is as a great teacher that Boerhaave remains, and will possibly always remain, in the popular mind”(Underwood 1977, 9), and in Lindeboom’s historiographic bias and selectivity.

In contrast, Cook judged that Boerhaave was an iconic and mythological figure and Williams doubted that Lindeboom’s work was “a serious critical study.” Their doubts warranted a more objective appraisal of Boerhaave’s clinical teaching.

In 1636, Leiden University’s Curators established a special clinical teaching facility, the *collegium medico practicum*, and, dividing one Saint Caecilia Gasthuis ward into two smaller six-bed wards, separated the female and male patients. Galleries behind each bed, and in the post-mortem theatre, allowed students to observe the professor’s examination of his patient’s and the surgeon’s post-mortems. Patients considered “suitable for demonstration to students,” (ibid, 145) were admitted to the *collegium* by the municipal doctors who, with other Saint Caecilia patients, received care from two appointed city doctors and a surgeon who performed the post-mortems (Beukers 1989, 143). Two professors were appointed to teach students the principles of practical medicine on Wednesdays and Saturdays in alternate nine-week periods in two eighteen-week semesters (Huisman 2008, 14; van Duijn 2020).

It was this original format of clinical instruction that Herman Boerhaave … followed after being appointed to carry out such “*practical exercise*” by the curators of Leyden University on August 8, 1714 … as it was carried out for twenty-four years. (Risse 1989, 1–2).

From 1714–1719, Boerhaave shared the clinical teaching with Frederik Dekkers as *collegium* co-professor from 1697 to 1719 (Lindeboom 1968b, 286), and “From 1820 Oosterdijk Schacht helped him with his task. Both clinicians took their turns, each lecturing in rotation” (*ibid*). Boerhaave commended Schacht in *Commentariolus* item XX111:

There was no need to look about for men to support and protect medical science other than … the very famous Oosterdijk, without doubt in years and work his equal, with his very thorough knowledge of science and his complete and quite incomparable experience. (*ibid*, 385)

And, when Boerhaave retired, “Oosterdijk Schacht took over his lectures” (*ibid*, 289).

In 1714 Boerhaave commenced his “*practical exercise*” *collegium clinical teaching* which was discontinued in 1723 due to illness and briefly resumed in 1737. But, as clinical teaching required a patient to be present, and as no patients were admitted into the *collegium* after 1723 (*vide infra*, 19–21), any teaching by Boerhaave after 1723 must have taken place in a non-clinical setting without patients. Therefore, any subsequent Boerhaave *praxis medica* teaching was not *clinical* teaching, as it was for his non-matriculant private attendees. For these reasons, Risse’s assertion that Boerhaave had carried out *clinical teaching* for twenty-four years is wrong.

Lindeboom, in asserting, “In the thirty-seven years of Boerhaave’s teaching he helped to educate a very great number of young men as physicians” (Lindeboom 1968b, 386), failed to distinguish between matriculant university students who would gain a Leiden MD and become physicians and the large numbers of fee-paying private attendees, some of whom may have graduated elsewhere. His further assertion that “Soon after his appearance as a lecturer, students who had been gradually moving away from Leiden began to return to it,” (Lindeboom 1968b, 356) was refuted by Harold Cook:

But as Willem Frijhoff has shown, the period of Boerhaave’s professorships shows a decline in both the absolute number of foreigners who took their medical degrees at Leiden and in the percentage of foreigners who studied at Leiden rather than at other Dutch Universities. (Cook 2000, 224; Frijhoff 1981)

Lindeboom further stated: “more students took their degree [under Boerhaave] than under any other medical professor at Leiden … except for anatomy and surgery [Boerhaave taught] all the subjects of the curriculum … and finally clinical medicine” (Lindeboom 1968b, 356). He then praised Boerhaave’s promotion of 178 PhD students between 1709–1738 (6 per year). However, the Leiden University Senate archives show that from 1717–1766, Bidloo, often absent as physician to William III, averaged a little over four PhD promotions per year, Dekkers averaged almost five, Albinus, the anatomist and surgeon professor, almost five, and Le Mort, the chemist, almost three per year. Kroon reported neither Schacht’s nor Boerhaave’s medical PhD promotions but noted that Boerhaave, as professor in two medical disciplines as well as botany and chemistry, “lectured on a greater number of subjects than other professors” (Kroon 1918, 292) thereby having the opportunity to teach the far greater number of students.

Lindeboom made two further assertions; “There is no doubt that Boerhaave was at his best at the bedside … [and] he did not discuss doctrines and systems, but devoted all his attention to signs and symptoms” (Lindeboom 1968b, 291). His first assertion, “There is no doubt that Boerhaave was at his best at the bedside,” was neither supported by Harm Beukers’ data, “Boerhaave’s pupils did not report that there were many clinical demonstrations in the Caecilia Hospital” (Beukers 1989, 147), nor by Haller’s report of only two demonstrations during his 1725–1727 studentship, nor by Swieten’s extant record of Boerhaave’s two clinical lectures in 1737, Case 1, Cachexy September 28, 1737, and Case 2, History of Palsy, September 28, 1737 (Boerhaave 1745a, 287–291). However Lindeboom stated that Swieten had recorded other cases in this period (*ibid*). Nor is it supported by the collegium admission data (*vide infra*, 19–21). The second assertion, “he did not discuss doctrines and systems, but devoted all his attention to symptoms and signs,” is also incorrect. Boerhaave’s limited duration yet famed clinical teaching in the *collegium practicum medica* was an observation-based method and an expository discourse in which he explicated on the theories, or the body of thought, or the doctrines that he recorded in his *Aphorismi.* His *clinical* demonstration-lectures each centred on a single selected patient whose history he presented and whose clinical signs he deduced through observation and explained through patho-physiological iatromechanical theories to establish her diagnosis and treatment. Boerhaave’s extant recorded teaching method included the same patient on two separate occasions in September 1737. Boerhaave’s method was not the “*practical exercise*” for which he was appointed that had been determined when the *collegium* was first established; rather his theoretically derived *Aphorismi* were the centrepiece of his lecture-demonstration teaching method in which he incorporated the new discoveries in medicine and natural philosophy into the accepted teaching of Hippocratic medicine (Knoeff 2010).

The second part of Lindeboom’s assertion was disproven by Bynum and Porter; “Historians have described the Leiden model, with small wards holding sample cases about whom practitioners didactically lectured to illustrate diseases, as ‘the proto-clinic.’… Like anatomical dissections in the medieval period, these cases primarily served to display the application of textual knowledge, rather than to offer students raw material for independent observations” (Bynum and Porter 1993, 1162). Gunter Risse also commented; “[Boerhaave’s] celebrated lectures at the hospital were meant to illustrate that a synthesis between theory and practice [supported] the notion that medical experience and book knowledge could be harmoniously integrated” (Risse 1989, 4–5).

Lindeboom’s immoderate assertions were matched by Underwood’s “how by his teaching at the St Caecilia Hospital … he introduced the modern method of clinical instruction which has remained the basis of medical education until the present day.” Underwood, a historian, disregarded Giovanni Monte’s introduction of ward-based clinical teaching when Paduan Professor of Practical Medicine in 1539, where, “students as a group accompanied their teacher to the bedside of the ill, both to observe their physician–teacher’s methods and to practice those methods under his observation and direction … where they observed the patient’s appearance, talked to her about her symptoms, checked the pulse, and observed everything necessary to determine the illness” (O’Malley 1970, 95–96).

Nor did Boerhaave teach as Franciscus Sylvius had as Leiden Professor of Medicine from 1658–1672:

I have led my pupils by the hand to medical practice, using a method unknown in Leiden, or perhaps elsewhere, that is, taking them daily to visit the sick at the public hospital. There I have put the symptoms of disease before their eyes; have let them hear the complaints of the patients, and have asked them their opinions as to the causes and rational treatment of each case, and the reasons for those opinions. Then I have given my own judgement on every point. Together with me they have seen the happy results of treatment when God has granted to our cares a restoration of health; or they have assisted in examining the body when the patient has paid the inevitable tribute to death. (White 2009, 771)

Lucas Schacht, 1670–1689 Leiden Professor of the Institutes of Medicine, gave this account of Sylvius’ teaching;

When he came with his pupils to the patient and began to teach, he appeared completely in the dark as to the causes or the nature of the affection the patient was suffering from, and at first expressed no opinion on the case; he then began by questions put to different members of his audience, to fish out everything and finally unite the facts discovered in this manner into a complete picture of the disease in such a way that the student received the impression that they had themselves made the diagnosis and not learnt it from him. (Puschmann 1891, 411)

Sylvius’ clinical instruction centred on teaching the essential practical semiotic skills of patient history taking and careful and thorough physical examination. Each requires, “both student and instructor to attend the patient’s bedside to discuss the case and/or demonstrate a clinical procedure” (Wojtczak 2003, n.p.).There exists no data to suggest that Boerhaave had undertaken such clinical instruction.

Nor did Boerhaave compare to Sylvius in the conduct of post-mortems. Sylvius’ student Robert Sibbald recorded twenty-three *collegium* post-mortems by Sylvius from 1660–1661, (Powers 2012,) and Boerhaave’s students, Burton, Haller, and Swieten made no reference to *collegium* post-mortems, nor were any mentioned by Suringar. The first to claim that Boerhaave had done so was Singer in 1928 (Singer 1928), a claim that Lindeboom was to affirm on three occasions without supporting data.

The only Boerhaave post-mortems records are those he published himself. Both were private aristocratic patients of Dr Samuel du Rij (Lindeboom 1968b), who consulted Boerhaave about his first patient’s diagnosis and management in 1723, and the second in 1727. In each the diagnosis was established at post-mortem. Boerhaave’s first patient was seen the night before he died, and the next day’s post-mortem revealed a ruptured oesophagus. Boerhaave’s case-notes, published in 1723, described the post-mortem findings in detail; “both physicians performed a post-mortem, assisted by an experienced servant, at which three other persons were present”(*ibid*). When translated into English in 1955 (Derbes and Mitchel 1955, 271–240), Lindeboom suggested that, “The history of a very interesting case gives an idea of Boerhaave’s private practice as well as a documentation of his clinical abilities” (Lindeboom 1968b, 153). The second, a case of mediastinal tumour was published by Boerhaave in Latin, French, and German in 1728 (Boerhaave 1728, 153), and translated into English in 1968 (Smith and King 1968, 331–348). While Boerhaave described his daily written communications with du Rij, the patient was first seen at post-mortem; “On the following day the very learned Du Ry, the skilled surgeon Porcher and I, in the presence of Arman Hardy de Vique, uncle of the deceased, prepared ourselves for the task ahead by washing the cadaver” (Boerhaave 1728, 111).

Neither post-mortem was performed by Boerhaave alone, and as no Saint Caecilia post-mortem records had been kept after the late seventeenth century (Beukers 1989, 142–143), the assertions by both Singer and Lindeboom that Boerhaave had performed *collegium* post-mortems are unsupported. Each overlooked that the attending Saint Caecelia surgeon was expected to perform post-mortems on both *collegium* and other Saint Caecelia wards deceased.

Lindeboom’s unsupported contentions caused Elisabeth Williams to conclude; “Lindeboom offered many assessments of Boerhaave’s importance that rested on little beyond assertion” (Williams 2009, 256), and Harold Cook to write: “Gerrit Lindeboom’s major English-language study of Boerhaave … is a revival of Schulten’s enthusiasm in modern dress” (Cook 2000, 222).

By employing such assertions in an endeavour to revive the heroic legend of Herman Boerhaave, Lindeboom misrepresented the nature of Boerhaave’s teaching by failing to distinguish between the intended practical exercises function of Boerhaave’s *collegium clinical* teaching appointment and his ongoing *non-clinical Aphorismi-*based lectures to private attendees*.* Lindeboom also misrepresented the duration of Boerhaave’s clinical teaching at the *collegium medico practicum* (Lindeboom 1968b).

## Part 4. Patient Admissions and Boerhaave’s Attendances at the Saint Caecilia *Collegium*

The registry of admissions to the *collegium* for the last half of the seventeenth century and from December 4, 1699 to January 12, 1753 (Cook 2000), gives reason to doubt that sufficient patients had been available for the teaching of practical medicine throughout much of the tenure of Boerhaave’s appointment. The patient admissions to the *collegium* from 1659–1661 numbered 160, from February 1684 to May 1685, ninety-nine, and from 1700–1710, the annual admissions averaged 43.4, peaking at eighty-seven in 1704. Thereafter, from 1711–1720, the admissions averaged 17.5, from 1720–1732, just three, and none thereafter until 1736 (*ibid*, 145–146).

In the first six years of Boerhaave’s *collegium* teaching, fewer than one patient was admitted each week to the *collegium* wards, and from 1720, if the *clinical* teaching of practical medicine had occurred, it would have depended on patients being brought from other Caecilia wards and, from 1722, taught by Oosterdijk Schacht. Alternately, it would have relied on practical medicine being taught without patients, as occurred in Boerhaave’s private *praxis medica* lectures.

Boerhaave’s university teaching was further compromised by illness in 1722–1723, 1727, 1729, 1731, and 1736. In each period Boerhaave was unable to teach for extended periods; in 1722 he was bedridden for almost six months, and, “Finally on 11 January 1723 he was able to resume his *private* lectures” (Lindeboom 1968b, 128). Boerhaave, in a 1723 letter to his friend Bassand, wrote; “But the expectations of both my patients and my pupils stand in the way of my wishes … let it be known that I wished to have nothing more to do with practicing medicine” (Knoeff 2010, 274). That Boerhaave did not resume his Sain Caecilia *collegium* ward rounds after recovering from his 1722–1723 illness was made evident in 1726 in a further letter in which he confirmed that he had, “retired from the turbulence of medical practice three years ago” (Lindeboom 1962, 239).

However, his non-clinical lectures, both to university students and the attendees at his remunerative private lectures, were not a part of his “medical practice,” for Boerhaave continued both his private and public non-clinical university lectures, resumed his private consultant practice for professional colleagues and nobility, and continued his considerable written consulting practice predominantly between he and his past students. In 1727, while confirming that his private consulting practice had continued, he made clear that he had ceased doing rounds; “Never have I had more patients to treat, although I have long since given up my practice (doing rounds)” (*ibid*, 251) and, in 1728, he apologized to Bassand for being unable to provide clinical experience for Bassand’s protégé (*ibid*, 253).

Given the negligible *collegium* admission numbers after 1720, Boerhaave’s recurrent illnesses and his correspondence, his acclaimed public ward rounds and famed bedside clinical teaching was limited to the nine-year period, 1714 to 1722, with a brief resumption in 1737 when patients were again admitted to the *collegium* wards, a period that included his two extant teaching case histories.

Given Lindeboom’s documentation of Boerhaave’s illnesses and his 1723 retirement, his assertion; “From 1714 to 1738, during nearly a quarter of a century, he carried on the clinical teaching faithfully and with enormous success”(Lindeboom 1968b, 296), is inconsistent with the moral and intellectual responsibility he assumed as a historiographer. Further, given the negligible *collegium* admissions, his nine-year collegium attendance and the appointment of a surgeon to perform the Saint Caecilia post-mortems, Lindeboom’s unsupported suggestion that, “the [*collegium*] clinicians themselves performed the post-mortem examinations. Boerhaave must have carried out many” is, at best, improper conjecture (*ibid*, 106).

Lindeboom’s assertions regarding Boerhaave’s clinical teaching led Rina Knoeff to suggest in 2010 that Suringar had laid the foundations for the legend that has characterized Boerhaave’s clinical teaching (Knoeff 2010) and to contend that Boerhaave’s clinical teaching was an heroic story.

Leiden clinical teaching is mainly associated with the name of Herman Boerhaave the famous Dutch medical teacher. The Leiden medical curriculum was very popular for its hands-on bedside teaching and that Boerhaave was the guiding spirit of a great increase in quality of the teaching. This heroic story, however, is a 19th-century invention. Rather than Boerhaave, it was Sylvius who was the champion of Leiden bedside teaching. During the 1660s and 1670s he applied Heurnius’ idea of diagnosing patients in discussion with students, and made hospital visits a part of the medical curriculum. Instead of only two days a week, Sylvius visited the hospital daily. He involved his students in his rounds and they made careful notes. Sylvius also regarded post-mortems as crucially important to clinical teaching. He taught that only through post-mortems could the “injured part and unnatural constitution” be demonstrated and diseases explained; his students were involved in hands-on medical teaching. (Knoeff 2012)

## Part 5. Assertions and Epithets as Vehicles of Historical Memory

Gerrit Lindeboom clearly believed that his accounts of Boerhaave’s contributions to clinical teaching were true, the acceptance of which rested on his reader’s trust and on his moral and intellectual responsibility as its author. That trust was identified by Henry Sigerist; “The writer of a biography re-creates a man. Our present medical students did not know William Osler … To them Osler is the man whom Harvey Cushing pictured” (Sigerist 1951, 33).

Beyond the assertions that have been considered and disproven, remains Lindeboom’s 1968a, b, c assertion that the *collegium* teaching preceding Boerhaave’s appointment had been so neglected (Lindeboom 1968b, 286) that Boerhaave’s 1714 appointment was, “to raise bedside teaching to unknown heights, and on it was to rest much of his immortality” (Knoeff 2010, 105–106). Again, Lindeboom’s assertion is false, for Beukers’ data demonstrated that the *collegium* admissions preceding Boerhaave’s appointment had exceeded those at any time during his tenure ( Beukers 1989, 85).

The progressive decline in admissions, Boerhaave’s recurrent illnesses, his discontinuation of ward rounds and an absence of student records of his clinical teaching cases before 1722, have discredited Lindeboom’s assertions. Nevertheless, Lindeboom’s assertions were sufficient to have his colleague Underwood attest; “how by his teaching at the Saint Caecilia Hospital at Leiden he introduced the modern method of clinical instruction which has remained the basis of medical education until the present day” (Underwood, 1968, xvi) and, to have Lord Cohen declare; “As a clinical teacher, he was unsurpassed in his age … Boerhaave certainly shone as a bedside teacher” (Cohen 1969, 408–409).

By endorsing Lindeboom’s heroic story of Boerhaave’s clinical teaching, both Underwood and Cohen encouraged other acolytes to accept Lindeboom’s Boerhaavian myth, some of whom were to perpetuate it epithetically. Perhaps neither medical historian was familiar with Henry Sigerist’s previous sage counsel; “The very popular hunting for ‘Fathers’ of every branch of medicine and every treatment is … rather foolish” (Sigerist 1951,13).

Burton, employed the first epithet, “The Batavian Hippocrates” in 1743, which Osler used in 1901 (Osler 1905, 230) and changed in 1907 to “The Dutch Hippocrates” (Osler 1907, 8). However, in likening Boerhaave to Hippocrates, both Burton and Osler implied that the clinical skills, writings and contributions to the practice of medicine by Boerhaave was comparable to that of Hippocrates. But Boerhaave’s *Institutiones* and his *Aphorismi* were based on his adaptations of other’s works and not on an extensive personal clinical experience, and each of his books were to be improved by the revisions published by Haller and Swieten. Whereas the Hippocratic books, *Aphorisms*, *Prognostics*, *Epidemics* and others, were based on its writers’ extensive clinical experiences and nosological observations, Boerhaave’s was based solely on his reading—as he wrote in the *Aphorismi’s* preface. Osler’s epithet was oratory hyperbole, while Burton’s reflected the excess of Schulten’s eulogy predicated on Boerhaave’s autobiographical notes.

The second epithet, Haller’s *Communis Europae Praeceptor*, “The Teacher of Europe,” is warranted, for through his public lectures to Leiden matriculants and private lectures to others from Great Britain and diverse European countries, his students became teachers in centres such as Vienna, Berlin, Gottingen, Uppsala, Edinburgh, and from there, Philadelphia.

In contrast, those that followed Lindeboom’s biography were inspired by his immoderate assertions, and include: “Father of Modern Medicine;” (Parker 2010, 69), “Father of teaching at the bedside” (Ellis 2018, 711), “Father of bedside teaching” (Kaiser 2006, 340), “Master of Bedside Teaching,” (Hull 1997, 512), and, the popular but unsourced, “Founder of Clinical Teaching.” The further epithet, “Modern medical science commenced with Boerhaave,” (Guthrie 1959, 112) preceded Lindeman’s biography.

These, with Underwood’s assertion, “[Boerhaave] introduced the modern method of clinical instruction which has remained the basis of medical education until the present day” (Underwood 1968, xvi), reflect a narrative of Boerhaave’s contributions to clinical teaching that has perpetuated the heroic status that Schultens initiated, which in turn was furthered by Burton, Suringar, and Lindeboom. Underwood’s assertion reflects his reliance on, and trust in, Lindeboom’s data that was to be accepted without question by each epithet author.

Underwood’s assertion that Boerhaave had, “introduced the modern method of clinical instruction” is wrong. Practical clinical teaching had existed in ancient civilizations (Sigerist 1951), in the Middle Ages (Riesman 1935), in the Islamic Caliphate hospitals (Sidek 2012), and in Padua under Taddeo Alderotti ~1210–1295 (Prioreschi 2003). Two hundred years later, Monte (1498–1551), as Professor of Practical Medicine, took students to the San Francesco where they received instructed in his methods of clinical examination and practiced such skills under his supervision. His method was adopted in many European medical schools and its introduction to Leiden was recommended by Johannes Heurnius (1543–1601). It was subsequently implemented by his son, Otto Heurnius (1577–1652), who taught the enthusiastic, innovative and highly regarded Franciscus Sylvius (1617–1672), who was followed by Govert Bidloo and, ultimately, Herman Boerhaave.

As authentic bedside clinical teaching had been taught in Leiden long before Boerhaave’s 1714 appointment as Professor of Practical Teaching, the suggestions that Boerhaave was the “Founder of Bedside Teaching” or the “Father of Teaching at the Bedside,” are manifestly untrue. The further epithet, “Master of Bedside Teaching” is also untrue. Despite Boerhaave being a skilled lecturer, he demonstrated none of skills that Monte and Sylvius had in their bedside clinical teaching. They had instructed students in the practical study of signs and symptoms, in the skills required in history taking and physical examination, and they observed their students in their practice of the skills that are inherent in the practice of the Art of clinical medicine. Boerhaave presented the students with the patient’s history and described her signs. The further epithet, “Father of Modern Medicine” that credits Boerhaave with the introduction of the system, or method, of modern medicine, and “Modern medical science commenced with Boerhaave” that claims Boerhaave introduced and integrated the sciences taught today as basic medical sciences are again untenable. Such assertions overlook that the subjects of anatomy, botany, semiotics and therapeutics, and chemistry added in 1669 when Care de Maets was appointed Lector, had, with bedside clinical teaching as the essence of the Art of Medicine, comprised the early Leiden curriculum taught throughout much of the seventeenth century. In its second half, the Institutes had been taught by Lucas Schacht and Charles Drelincourt, and, in 1655, Lazarus Riverius published *Institutiones Medicae* in 1644 that contained chapters on physiology, pathology, semiotics, therapeutics and hygiene, that preceded Boerhaave’s *Institutiones Medicae* and *Praxis Medica* (King 1958, 65–81), and both Friederich Hoffmann, in *Medicinae rationalis systematicae* (Halle 1718–1720), and George Ernst Stahl in *Theoria medica vera, physiologiam et pathologiam, tanquam doctrinae medicae partes vere contemplativas, e naturae & artis veris fundamentis* (Halle 1708) had previously published their conflicting theories of physiology, iatromechanics, iatrochemistry, and semiotics.

Equally untrue is Cohen’s assertion; “As a clinical teacher, he was unsurpassed in his age. He taught a class of one hundred students … for five hours a day, four days a week.” Cohen, in doing so, failed to distinguish between *clinical* and *non-clinical* teaching. The teaching Cohen referred to were the Institutes lectures that Boerhaave gave throughout his career to matriculant university students and to those who attended his highly popular private classes. Such lectures were neither at Saint Caecilia, nor clinical as was defined above. Cohen’s assertion reflects his iconolatry and his misunderstanding of the essence of clinical teaching.

His further assertion, “Boerhaave certainly shone as a bedside teacher” (Cohen 1969, 408–409) reflects the excess of an admirer and believer who was less than objective in reviewing Lindeboom’s *Herman Boerhaave*. In undertaking his review, Cohen must have read of Boerhaave’s recurrent illnesses, his letters to Bassand and his discontinuation of clinical teaching rounds in 1723.

Such apocryphal epithets and assertions reflect a hyperbole that sought to sustain the myths of Boerhaave’s bedside clinical teaching method that: he taught students at the bedside for twenty-four years; had performed *collegium* post-mortems; had introduced a method of teaching that remains in clinical teaching, and had increased Leiden’s medical student numbers. While such claims have been shown to be historically unreliable, Lindeboom’s narrative of Boerhaave’s personal life and his many contributions to Leiden University, to the fields of chemistry and botany, and his service to the wider medical community remain just and warranted.

Lindeboom was an established academic historiographer who could have been expected to have understood the ethical standards required of historical writing, including the need to ensure the reliability of data presented. Instead, he provided a subjective interpretation based on a highly selective subset of the data, omitting evidence that was inconsistent with his preferred narrative. By obscuring the idiosyncratic and contestable elements of his interpretation in this way, he thereby failed to observe a key ethical responsibility underlying scholarly discourse.

Historiographers in general are bound by an inherent responsibility to appraise critically all available data and both to declare transparently and to justify cogently any value judgements on which they rely. They are obliged in this manner to protect the trustworthiness of their data and the interpretations attached to them, and to avoid the possibility of compromise associated with personal biases, sympathies, and presuppositions. While Underwood, Cohen, and others undoubtedly accepted in good faith the trustworthiness of Lindeboom’s narratives our investigations have sadly raised serious questions about the validity of the conclusions he sought to aver.

## Part 6. An Epilogue: The Facilitation of Historical Error Through Apocrypha

Harold Cook determined that Boerhaave was an iconic and mythological figure whose standing was magnified by the hyperbolic praise that attributed to him innovations that were not his, while data that disproved such assertions was overlooked (Cook 2000, 221–240). Cook, in citing Frijhoff, suggested that the seventeenth century changes in Dutch academic medicine had determined the Leiden medical curriculum that advanced eighteenth century medical education, enabling Boerhaave’s personal genius and his Professorships to made him “a Faculty in himself” (Lindeboom 1968b, 122) and equipping him to lecture in the way he had.

Lindeboom put aside the advances made by Leiden University in medical education during the seventeenth century in his determination to revive and enliven Herman Boerhaave’s reputation and attribute to him advances that were not his. By ignoring the successive cautions expressed by Haller, Cullen, Pettigrew, Allbutt, Buck, Riesman, and Daremberg and selectively using the data published by Kroon, Lindeboom shifted the function of medicine’s historical writing from an objective and reliable formulation to one in which hyperbole and unfounded assertions were presented as truths, reflecting that which Sigerist had determined; “The history of the physician has sometimes been distorted when … written by doctors who had not sufficient historical training” (Sigerist 1951, 15).

Lindeboom’s erroneous historiography was accepted by others who perpetuated his unsubstantiated and apocryphal assertions as historical truths. Boerhaave neither made original contributions to clinical teaching nor did he institute or methodise Leiden’s well-established Institutes curriculum; he was not the Father of Bedside Teaching. The myth and iconolatry within Lindeboom’s historiography caused Johnsen’s doubts to be overlooked, led Singer to assert that Boerhaave had performed post-mortems at the Saint Caecilia *collegium*, and left Suringar alone in recognizing that the Saint Caecilia’s *collegium practicum-medica* teaching was undertaken by both Herman Boerhaave and Oosterdijk Schacht. Notably, it was Williams who recognized the personal inspiration that Lindeboom gained from his studies and writings about Boerhaave.

Boerhaave was an exceptionally skilled communicator and learned theorist, whose heutagogic study of medicine precluded his practical instruction in the long accepted and critically important clinical discipline of semiotics. Boerhaave’s oratory skills enabled him to elaborate on his individual observational-based clinical findings and to apply his iatromechanical pathophysiological theories to determine a diagnosis and formulate therapy. Boerhaave did not teach the practical semiotic clinical skills that others such as Sylvius had before him at Leiden.

While his theories, texts, and teaching methods that centred on the works and methods of Hippocrates and Sydenham, were accepted widely in Europe, Edinburgh, and Philadelphia, Boerhaave made no claim that his teaching methods were innovative. Rather it was his students who improved his books who did so and furthered a reputation that was magnified by Suringar and elaborated by Lindeboom. Despite his theories and methods being supplanted as physiology and medical science advanced and new textbooks such as Cullen’s 1775 *First Lines* were published, Herman Boerhaave remains a deserving hero of Dutch medicine whose life and contributions were celebrated in the 1938 international bi-centenary of his death.

The heroic story of Herman Boerhaave’s clinical teaching was fundamental to Lindeboom’s iconolatry, and his assertions, though fallacious, inspired hero-worshiping, a band-wagon of literature and many false epithets. In misrepresenting the history of clinical teaching at Leiden University, Gerrit Lindeboom has exemplified the error of history that William Dunning wrote of in 1914; “history is determined no more by what is true than by what men believe to be true” (Dunning 1914, 2).

## Data Availability

The data generated and used in this study are available from the sources referred to in the text and the References.

## Footnotes

1 Boerhaave’s lifelong motto: “Simplicity is the sign of truth.”
